# Supplementary material for: Depth moderates loss of marine foundation species after an extreme marine heatwave: could deep temperate reefs act as a refuge?
Source: Proc Biol Sci. 2020 Jun 10;287(1928):20200709. doi: 10.1098/rspb.2020.0709 (PMC7341917; doi:10.1098/rspb.2020.0709)
Supplement: Figure and tables [file rspb20200709supp1.pdf]

Electronic supplementary material

Depth moderate loss of marine foundation species after an extreme marine heatwave: Could deep temperate reefs act as refuge?

Ana Giraldo-Ospina [1,2*], Gary A. Kendrick [1,2], Renae K. Hovey [1,2]

[1] School of Biological Sciences, University of Western Australia, 35 Stirling Highway, Crawley, WA 6009, Australia

[2] Oceans Institute, The University of Western Australia, 64 Fairway, Crawley, Western Australia 6009, Australia

Supplementary table S1. Number of grids surveyed and used for analyses at each year for all sites and locations (ND = No Data, sites not surveyed that year).

	Site	Depth	2010	2011	2012	2013	2014	2017
**Abrolhos Islands**	***Geebank***	*15 m*	3	3	3	3	3	3
		25 m	3	3	3	2	2	3
	Coral Patches	*15 m*	3	2	3	3	3	ND
		40 m	3	ND	3	3	1	3
	Snapper Bank	*25 m*	ND	3	3	3	3	3
**Jurien Bay**	***North***	*15 m*	3	2	2	3	ND	ND
		25 m	3	2	2	3	ND	ND
		40 m	3	3	3	ND	ND	ND
	South	*40 m*	3	3	3	3	ND	ND
	New	*25 m*	*ND*	3	3	ND	ND	ND
**Rottnest Island**	***North***	*15 m*	3	3	3	3	ND	3
		25 m	3	2	2	3	ND	3
		40 m	3	2	2	3	ND	3
	South	*15 m*	3	2	2	2	ND	3
		25 m	3	3	3	3	ND	3
		40 m	3	2	2	2	ND	2

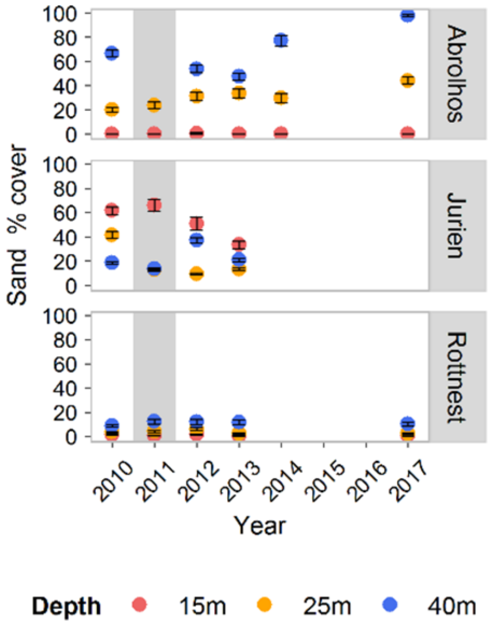

Supplementary figure S1. Mean percent cover (± SE) of sand at each depth and location from 2010 - 2017. The estimates of percent cover are means of ~ 90 to 180 images within each depth per year.

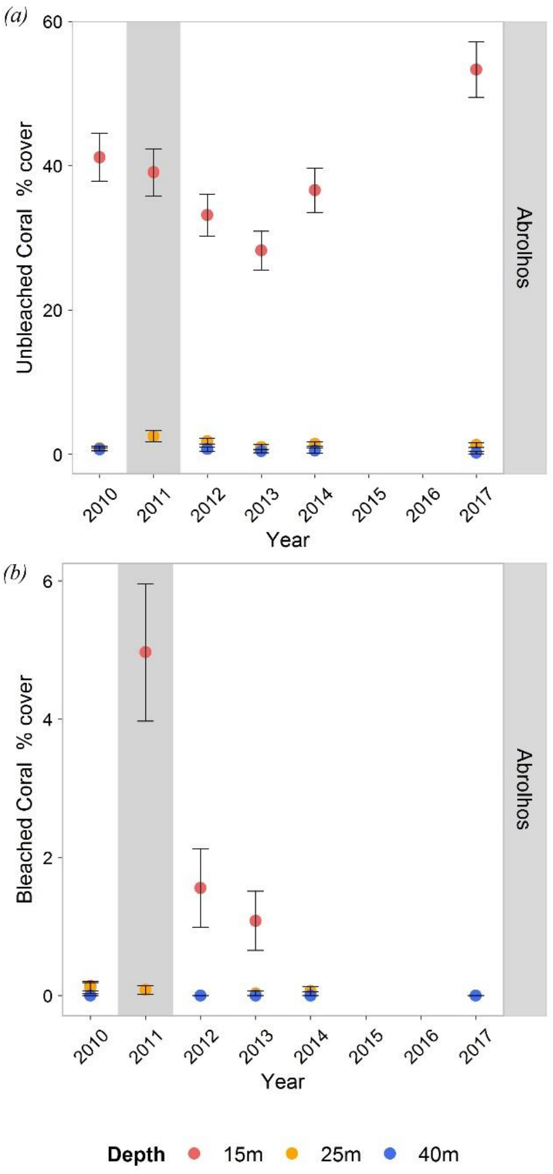

Supplementary figure S2: Mean percent cover (± SE) of unbleached coral (a) and bleached coral (b) at each depth at Houtman Abrolhos Islands, WA. The grey box highlights 2011, the year of the marine heatwave. The estimates of percent cover are means of ~ 90 to 180 images within each depth per year.

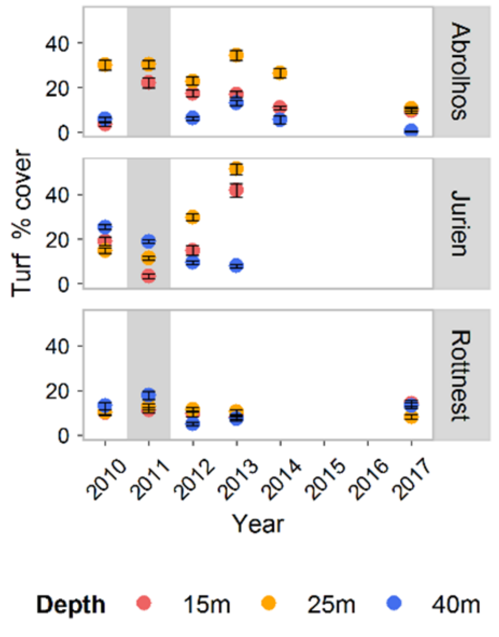

Supplementary figure S3. Mean percent cover (± SE) of turf at each depth and location from 2010 - 2017. The estimates of percent cover are means of ~ 90 to 180 images within each depth per year.

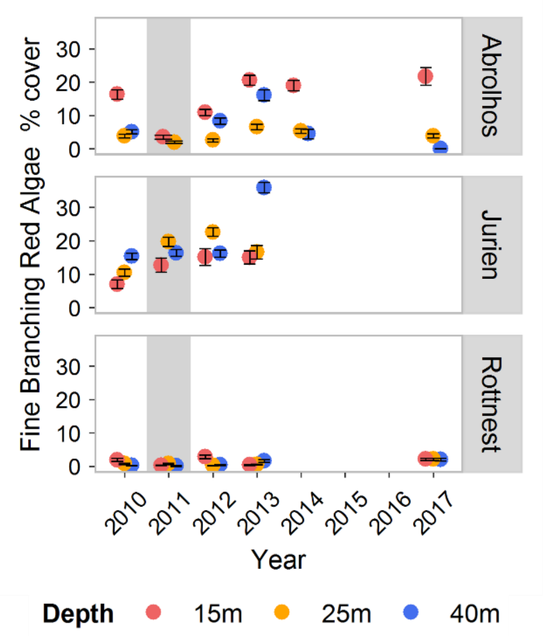

Supplementary figure S4. Mean percent cover (± SE) of fine branching red algae at each depth and location from 2010 - 2017. The estimates of percent cover are means of ~ 90 to 180 images within each depth per year

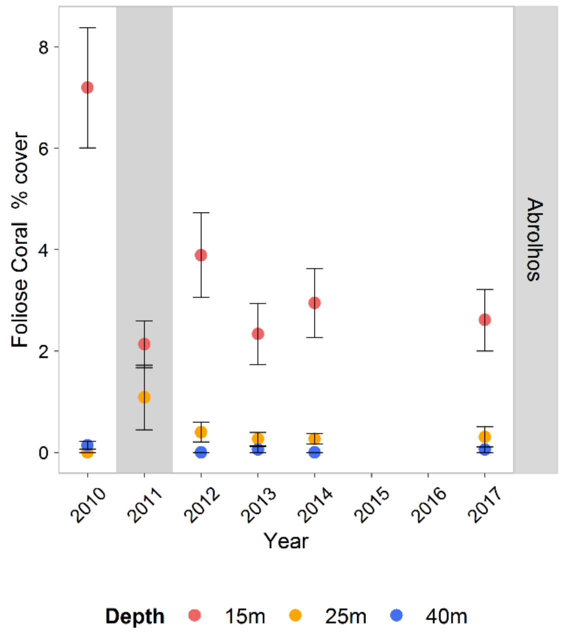

Supplementary figure S5. Mean percent cover (± SE) of Foliose Coral at each depth at Houtman Abrolhos Islands, WA. The estimates of percent cover are means of ~ 90 to 180 images within each depth per year.

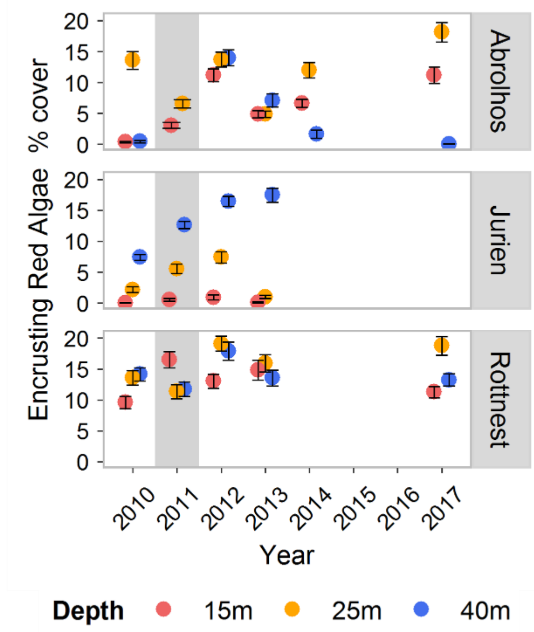

Supplementary figure S6. Mean percent cover (± SE) of encrusting red algae at each depth and location from 2010 - 2017. The estimates of percent cover are means of ~ 90 to 180 images within each depth per year.

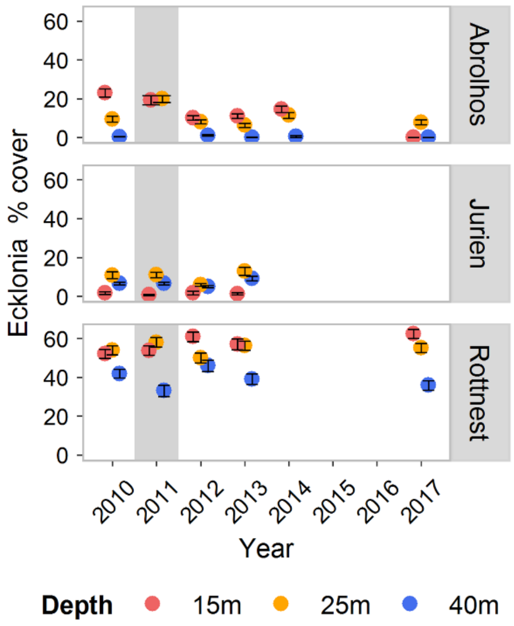

Supplementary figure S7. Mean percent cover (± SE) of *Ecklonia radiata* at each depth and location from 2010 - 2017. The estimates of percent cover are means of ~ 90 to 180 images within each depth per year.

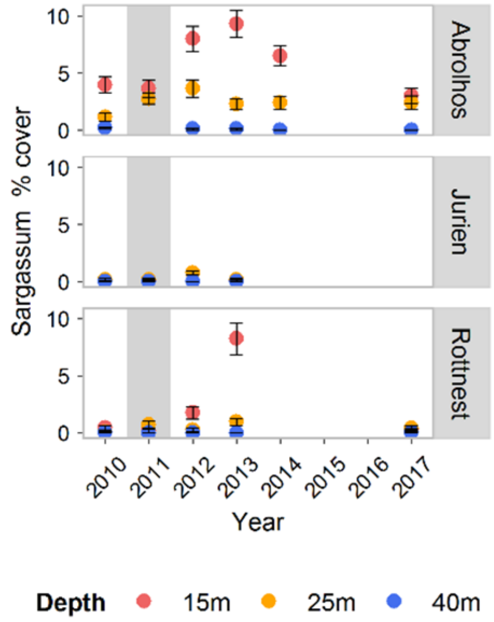

Supplementary figure S8. Mean percent cover (± SE) of *Sargassum sp*. at each depth and location from 2010 - 2017. The estimates of percent cover are means of ~ 90 to 180 images within each depth per year.

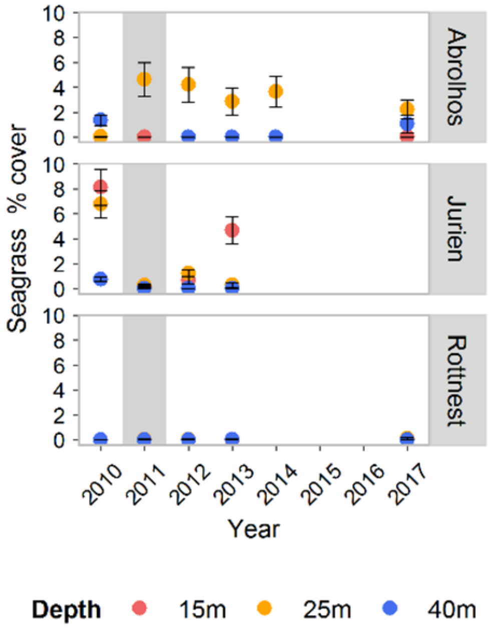

Supplementary figure S9. Mean percent cover (± SE) of seagrass at each depth and location from 2010 - 2017. The estimates of percent cover are means of ~ 90 to 180 images within each depth per year.

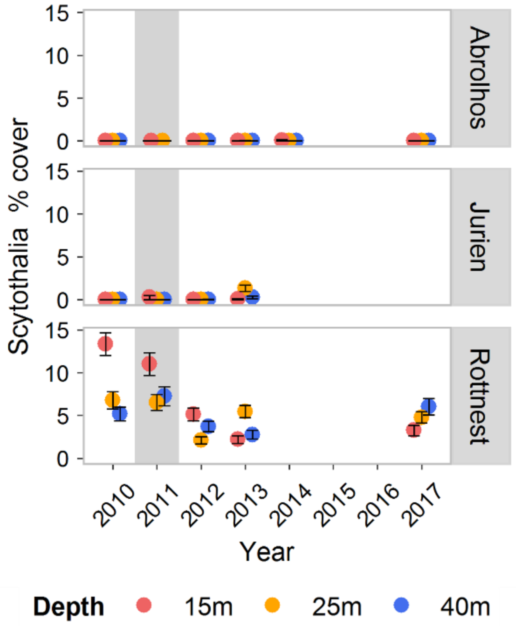

Supplementary figure S10. Mean percent cover (± SE) of *Scytothalia dorycarpa* at each depth and location from 2010 - 2017. The estimates of percent cover are means of ~ 90 to 180 images within each depth per year

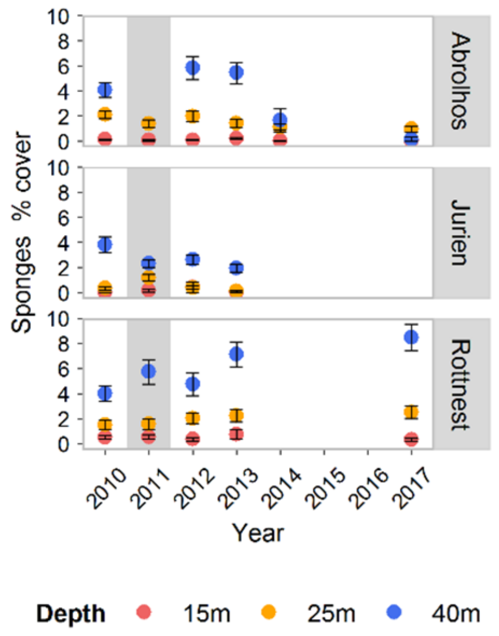

Supplementary figure S11. Mean percent cover (± SE) of sponges at each depth and location from 2010 - 2017. The estimates of percent cover are means of ~ 90 to 180 images within each depth per year.

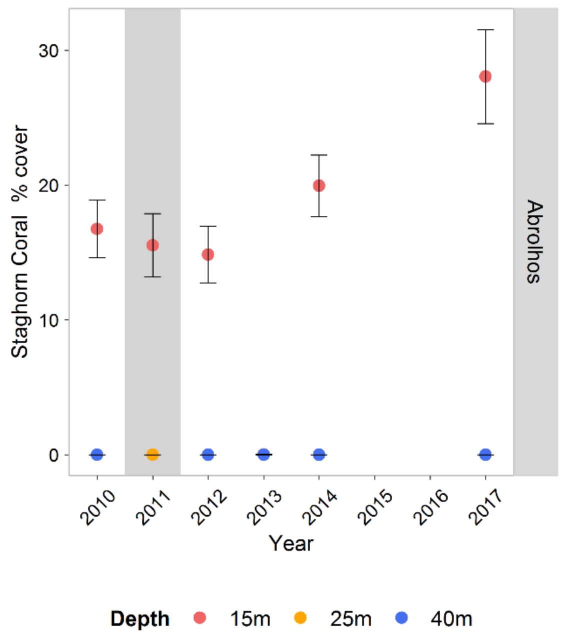

Supplementary figure S12. Mean percent cover (± SE) of staghorn coral at each depth and location from 2010 – 2017. The estimates of percent cover are means of ~ 90 to 180 images within each depth per year.

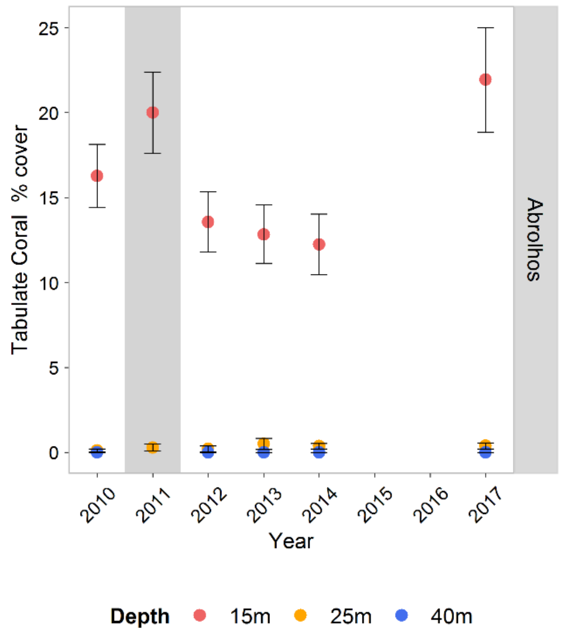

Supplementary figure S13. Mean percent cover (± SE) of tabulate coral at each depth and location from 2010 – 2017. The estimates of percent cover are means of ~ 90 to 180 images within each depth per year.

Supplementary table S2. Summary statistics of one-way ANOVA to test for the effect of year in the percent cover of *E. radiata*, turf, encrusting red algae and *S. dorycarpa*, at each depth and location. Values in bold show significant differences between treatment plots. Values marked with * denote non-parametric tests (Kruskal-Wallis chi-squared values). Tukey tests were performed when ANOVAs were significant, and Dunn's test when Kruskal-Wallis tests were significant.

Class	*Location*	*Depth*	*df*	*F-value*	*p-value*	*Post-hoc*
Ecklonia radiata	Abrolhos	15 m	2	0.29732 *	0.8619	
		25 m	2	0.725	0.502	
		40 m	2	2.343	0.158	
	Jurien	15 m	2	0.45226 *	0.7976	
		25 m	2	0.023	0.978	
		40 m	2	0.0042 *	0.9979	
	Rottnest	15 m	2	1.131	0.351	

		25 m	2	0.162	0.852	
		40 m	2	0.581	0.575	
**Turf**	Abrolhos	15 m	2	7.637	**<0.01**	**2010-2011**<br>2010-2014<br>**2011-2014**
		25 m	2	1.952	0.179	
		40 m	2	0.265	0.774	
	Jurien	15 m	2	2.439	0.149	
		25 m	2	28.17	**<0.001**	2010-2011<br>**2010-2013**<br>**2011-2013**
		40 m	2	10.017 *	**<0.01**	2010-2011<br>**2010-2013**<br>2011-2013
	Rottnest	15 m	2	0.792	0.472	
		25 m	2	1.334	0.295	
		40 m	2	0.38	0.692	
**Encrusting Red**	Abrolhos	15 m	2	10.675 *	**<0.01**	2010-2011<br>**2010-2014**<br>**2011-2014**
		25 m	2	1.128	0.351	
		40 m	2	7.799	**<0.05**	**2010-2012**<br>2010-2014<br>2012-2014
	Jurien	15 m	2	4.6012 *	0.1002	
		25 m	2	1.996	0.186	
		40 m	2	11.26	**<0.01**	**2010-2011**<br>**2010-2013**<br>2011-2013
	Rottnest	15 m	2	1.583	0.24	
		25 m	2	0.884	0.435	
		40 m	2	0.47	0.636	
Scytothalia dorycarpa	Rottnest	15 m	2	4.262	**<0.05**	2010-2011<br>**2010-2017**<br>2011-2017
		25 m	2	0.241	0.789	

		40 m	2	0.279	0.761

Supplementary table S3. Univariate PERMANOVA to test for percent cover differences of main benthic categories between depths and years at each location. PERMANOVA derived from square root transformation and Euclidian distance similarity matrices.

Benthic category	Location	Effects	Numerator df	Denominator df	Pseudo -F	p	Unique permutations
Coral	*Abrolhos*	Year	5	2413	5.09	**0.0002**	9963
		Depth^	2	2413	622.48	**0.0001**	9945
		Year x Depth	9	2413	5.98	**0.0001**	9939
Bleached Coral	*Abrolhos*	Year	5	2413	26.131	**0.0001**	9948
		Depth^	2	2413	33.299	**0.0001**	9947
		Year x Depth	9	2413	14.697	**0.0001**	9933
Coral - Staghorn	*Abrolhos*	Year	5	2413	16.13	**0.0001**	9941
		Depth	2	2413	335.11	**0.0001**	9948
		Year x Depth	9	2413	10.68	**0.0001**	9941
Coral - Foliose	*Abrolhos*	Year	5	2413	2.26	**0.0443**	9925
		Depth	2	2413	94.63	**0.0001**	9943
		Year x Depth	9	2413	4.14	**0.0001**	9926
Coral - Tabulate	*Abrolhos*	Year	5	2413	3.13	**0.0072**	9951
		Depth	2	2413	343.65	**0.0001**	9953
		Year x Depth	9	2413	3.27	**0.0006**	9931
Macroalgae	*Abrolhos*	Year	5	2413	25.329	**0.0001**	9840
		Depth	2	2413	116.37	**0.0001**	9941

		Year x Depth	9	2413	14.87	**0.0001**	9838
	Rottnest	Year	4	2355	5.385	**0.0006**	9963
		Depth	2	2355	198.2	**0.0001**	9949
		Year x Depth	8	2355	3.013	**0.0014**	9935
	Jurien	Year	3	1548	69.04	**0.0001**	9936
		Depth	2	1548	159.97	**0.0001**	9949
		Year x Depth	6	1548	25.09	**0.0001**	9953
Macroalgae - Ecklonia	*Abrolhos*	Year	5	2413	19.808	**0.0001**	9952
		Depth	2	2413	62.225	**0.0001**	9940
		Year x Depth	9	2413	8.821	**0.0001**	9947
	Rottnest	Year	4	2355	1.2873	0.2763	9958
		Depth	2	2355	70.477	**0.0001**	9945
		Year x Depth	8	2355	3.355	**0.001**	9938
	Jurien	Year	3	1548	4.865	**0.002**	9942
		Depth	2	1548	71.479	**0.0001**	9943
		Year x Depth	6	1548	2.333	**0.0297**	9939
Macroalgae - Sargassum	*Abrolhos*	Year	5	2413	5.419	**0.0001**	9953
		Depth	2	2413	77.164	**0.0001**	9955
		Year x Depth	9	2413	3.820	**0.0003**	9948
	Rottnest	Year	4	2355	36.549	**0.0001**	9958
		Depth	2	2355	55.544	**0.0001**	9951
		Year x Depth	8	2355	26.901	**0.0001**	9952
Macroalgae - Turf	*Abrolhos*	Year	5	2413	34.008	**0.0001**	9947
		Depth	2	2413	125.68	**0.0001**	9954

		Year x Depth	9	2413	12.888	**0.0001**	9828
	Rottnest	Year	4	2355	7.973	**0.0001**	9954
		Depth	2	2355	0.580	**0.5566**	9950
		Year x Depth	8	2355	4.956	**0.0001**	9926
	Jurien	Year	3	1548	64.997	**0.0001**	9940
		Depth	2	1548	49.855	**0.0001**	9944
		Year x Depth	6	1548	83.203	**0.0001**	9943
Macroalgae – Fine branching red	*Abrolhos*	Year	5	2413	46.374	**0.0001**	9948
		Depth	2	2413	182.78	**0.0001**	9949
		Year x Depth	9	2413	16.015	**0.0001**	9941
	Rottnest	Year	4	2355	18.362	**0.0001**	9957
		Depth	2	2355	8.238	**0.0002**	9947
		Year x Depth	8	2355	8.959	**0.0001**	9940
	Jurien	Year	3	1548	39.354	**0.0001**	9952
		Depth	2	1548	67.283	**0.0001**	9954
		Year x Depth	6	1548	17.338	**0.0001**	9935
Macroalgae – Encrusting red	*Abrolhos*	Year	5	2413	45.266	**0.0001**	9956
		Depth	2	2413	70.205	**0.0001**	9946
		Year x Depth	9	2413	21.739	**0.0001**	9919
	Rottnest	Year	4	2355	7.499	**0.0001**	9933
		Depth	2	2355	5.726	**0.0034**	9948
		Year x Depth	8	2355	4.596	**0.0001**	9935
	Jurien	Year	3	1548	39.292	**0.0001**	9954

		Depth	2	1548	643.85	**0.0001**	9960
		Year x Depth	6	1548	14.646	**0.0001**	9936
Macroalgae - Scytothalia	*Rottnest*	Year	4	2355	18.688	**0.0001**	9943
		Depth	2	2355	5.664	**0.037**	9955
		Year x Depth	8	2355	8.291	**0.0001**	9942
Macroalgae – Canopy	*Abrolhos*	Year	5	2413	11.552	**0.0001**	9949
		Depth	2	2413	88.42	**0.0001**	9953
		Year x Depth	9	2413	7.085	**0.0001**	9947
	Rottnest	Year	4	2355	0.365	0.8378	9944
		Depth	2	2355	78.439	**0.0001**	9945
		Year x Depth	8	2355	2.499	**0.0097**	9940
	Jurien	Year	3	1548	6.587	**0.0003**	9948
		Depth	2	1548	45.515	**0.0001**	9940
		Year x Depth	6	1548	5.488	**0.0001**	9936
Seagrass	*Abrolhos*	Year	5	2413	0.688	0.6219	9945
		Depth	2	2413	31.811	**0.0001**	9959
		Year x Depth	9	2413	3.675	**0.0003**	9940
	Jurien	Year	3	1548	33.929	**0.0001**	9949
		Depth	2	1548	22.252	**0.0001**	9952
		Year x Depth	6	1548	6.687	**0.0001**	9945
Sponges	*Abrolhos*	Year	5	2413	20.268	**0.0001**	9933
		Depth	2	2413	108.89	**0.0001**	9964
		Year x Depth	9	2413	11.53	**0.0001**	9931
	Rottnest	Year	4	2355	4.562	**0.0012**	9952
		Depth	2	2355	129.99	**0.0001**	9954

		Year x Depth	8	2355	2.774	**0.0040**	9927
	Jurien	Year	3	1548	1.292	0.2752	9952
		Depth	2	1548	93.322	**0.0001**	9951
		Year x Depth	6	1548	1.566	0.1512	9949
Sand	*Abrolhos*	Year	5	2413	19.23	**0.0001**	9944
		Depth	2	2413	1067.2	**0.0001**	9949
		Year x Depth	9	2413	7.921	**0.0001**	9929
	Rottnest	Year	4	2355	5.36	**0.0002**	9956
		Depth	2	2355	172.58	**0.0001**	9951
		Year x Depth	8	2355	1.122	0.3452	9925
	Jurien	Year	3	1548	17.509	**0.0001**	9953
		Depth	2	1548	147.03	**0.0001**	9953
		Year x Depth	6	1548	32.323	**0.0001**	9935

Supplementary figure S14. Temperature data series (in degrees Celsius) for the yearly summer (December – April) averages (a), and yearly anomalies (b) at each location and for average depth of 10 m (5 -15 m range), 30 m (25 – 35 m depth range) and 40 m (35 – 45 m depth range). Anomalies calculated over the 20-year period averages. The grey line highlights 2011, the year of the marine heatwave. Temperature data was used to identify the temperature signal of the marine heatwave at each of the locations and across the three target depths. Data was gathered from the Australian Shelf Temperature Data Atlas which groups temperature data collected from 1995 to 2014 from a range of sources including AUV deployments, Argo profiles, glider deployments, moorings, and CTD casts (Integrated Marine Observing System, 2015). Data within a 30 km radius from the study locations was selected for the analysis (see electronic supplementary tables S4-S6 for details of temperature data). The average temperature for the summer months (December – April) was calculated for each year (1995-2014) at each location and depth. Temperature data was not available for all years and months for Abrolhos and Jurien Bay, yet, the average summer temperature was calculated over the 20-year period and temperature anomalies were calculated over this average.

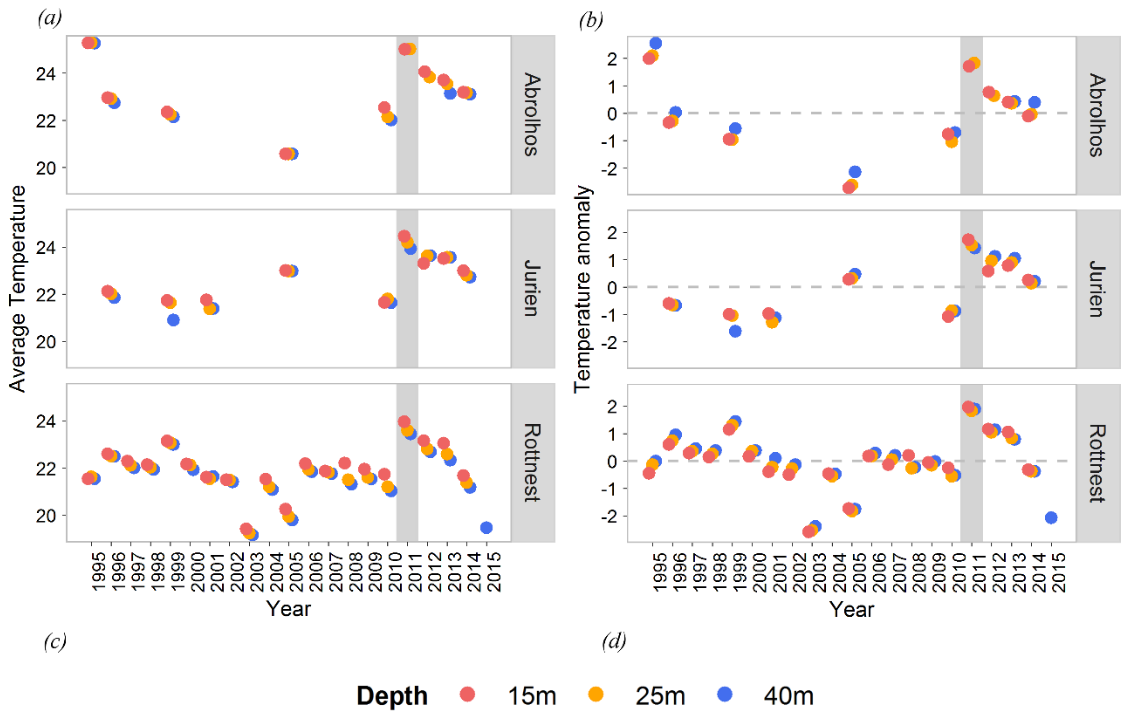

Supplementary table S4. Summer average temperatures in degrees Celsius (± standard error) for each location at three depth bins 10, 30 and 40 m from 1995 to 2015 (Integrated Marine Observing System, 2015). Depth bins group data from a range of depths so that 10 m bin include 5-15 m, the 30 m bin include 25-35 m and the 40 m bin includes 35-45 m of depth. Summer months included the temperatures from December to April. The standard error of some temperatures was not calculated due to lack of data (one or 2 observations only). ND indicates that there is no data available for that location and depth.

Year	Location	10 m	30 m	40 m
1995	*Abrolhos*	25.286 ± 0.064	25.298 ± 0.052	25.267 ± 0.033
1996	*Abrolhos*	22.96 ± 0.257	22.916 ± 0.286	22.739 ± 0.361
1997	*Abrolhos*	ND	ND	ND
1998	*Abrolhos*	ND	ND	ND
1999	*Abrolhos*	22.346	22.237	22.147
2000	*Abrolhos*	ND	ND	ND
2001	*Abrolhos*	ND	ND	ND
2002	*Abrolhos*	ND	ND	ND
2003	*Abrolhos*	ND	ND	ND
2004	*Abrolhos*	ND	ND	ND
2005	*Abrolhos*	20.574	20.575	20.569
2006	*Abrolhos*	ND	ND	ND
2007	*Abrolhos*	ND	ND	ND

2008	*Abrolhos*	ND	ND	ND
2009	*Abrolhos*	ND	ND	ND
2010	*Abrolhos*	22.537	22.151	22.007
2011	*Abrolhos*	25.008	25.024	ND
2012	*Abrolhos*	24.067	23.833	ND
2013	*Abrolhos*	23.705	23.551	23.138
2014	*Abrolhos*	23.193 ± 0.09	23.16 ± 0.074	23.106 ± 0.064
1995	*Jurien*	ND	ND	ND
1996	*Jurien*	22.127 ± 0.199	22.014 ± 0.22	21.847 ± 0.278
1997	*Jurien*	ND	ND	ND
1998	*Jurien*	ND	ND	ND
1999	*Jurien*	21.734	21.629	20.905
2000	*Jurien*	ND	ND	ND
2001	*Jurien*	21.762	21.38	21.388
2002	*Jurien*	ND	ND	ND
2003	*Jurien*	ND	ND	ND
2004	*Jurien*	ND	ND	ND
2005	*Jurien*	23.011	22.984	22.976
2006	*Jurien*	ND	ND	ND
2007	*Jurien*	ND	ND	ND
2008	*Jurien*	ND	ND	ND
2009	*Jurien*	ND	ND	ND
2010	*Jurien*	21.653 ± 0.695	21.801 ± 0.12	21.632 ± 0.31
2011	*Jurien*	24.459 ± 0.342	24.201 ± 0.333	23.934 ± 0.181
2012	*Jurien*	23.31 ± 0.23	23.622 ± 0.151	23.63 ± 0.166
2013	*Jurien*	23.507 ± 0.21	23.562 ± 0.219	23.557 ± 0.136
2014	*Jurien*	22.986 ± 0.247	22.802 ± 0.255	22.717 ± 0.298
1995	*Rottnest*	21.547 ± 0.418	21.626 ± 0.422	21.537 ± 0.416
1996	*Rottnest*	22.599 ± 0.245	22.502 ± 0.239	22.498 ± 0.245
1997	*Rottnest*	22.288 ± 0.413	22.114 ± 0.42	22 ± 0.469
1998	*Rottnest*	22.142 ± 0.25	22.026 ± 0.275	21.931 ± 0.288
1999	*Rottnest*	23.141 ± 0.287	23.064 ± 0.298	22.994 ± 0.296
2000	*Rottnest*	22.165 ± 0.402	22.121 ± 0.455	21.928 ± 0.515
2001	*Rottnest*	21.609 ± 0.169	21.537 ± 0.159	21.642 ± 0.195
2002	*Rottnest*	21.505	21.486	21.409
2003	*Rottnest*	19.413 ± 0.167	19.238 ± 0.282	19.162 ± 0.36
2004	*Rottnest*	21.53 ± 0.272	21.202 ± 0.167	21.078 ± 0.139
2005	*Rottnest*	20.258 ± 0.366	19.937 ± 0.275	19.792 ± 0.223
2006	*Rottnest*	22.177 ± 0.407	21.937 ± 0.343	21.838 ± 0.305
2007	*Rottnest*	21.865	21.813	21.754
2008	*Rottnest*	22.201 ± 0.845	21.497 ± 0.81	21.314 ± 0.835
2009	*Rottnest*	21.95 ± 0.114	21.603 ± 0.162	21.528 ± 0.174
2010	*Rottnest*	21.74 ± 0.211	21.207 ± 0.221	21.023 ± 0.243
2011	*Rottnest*	23.961 ± 0.184	23.585 ± 0.225	23.447 ± 0.232
2012	*Rottnest*	23.159 ± 0.28	22.807 ± 0.254	22.685 ± 0.255
2013	*Rottnest*	23.05 ± 0.144	22.594 ± 0.173	22.332 ± 0.2
2014	*Rottnest*	21.681 ± 0.3	21.382 ± 0.314	21.171 ± 0.331
2015	*Rottnest*	ND	ND	19.473

Supplementary table S5. Summer temperatures anomalies in degrees Celsius for each location at three depth bins 10, 30 and 40 m from 1995 to 2015 (Integrated Marine Observing System, 2015). Depth bins group data from a range of depths so that 10 m bin include 5-15 m, the 30 m bin include 25-35 m and the 40 m bin includes 35-45 m of depth. Summer months included the temperatures from December to April. ND indicates that there is no data available for that location and depth.

		**15 m**	**25 m**	**40 m**
Year	***Location***	***Anomaly***	***Anomaly***	***Anomaly***
1995	*Abrolhos*	1.989	2.104	2.557
1996	*Abrolhos*	-0.338	-0.278	0.029
1997	*Abrolhos*	ND	ND	ND
1998	*Abrolhos*	ND	ND	ND
1999	*Abrolhos*	-0.952	-0.957	-0.563
2000	*Abrolhos*	ND	ND	ND
2001	*Abrolhos*	ND	ND	ND
2002	*Abrolhos*	ND	ND	ND
2003	*Abrolhos*	ND	ND	ND
2004	*Abrolhos*	ND	ND	ND
2005	*Abrolhos*	-2.723	-2.619	-2.142
2006	*Abrolhos*	ND	ND	ND
2007	*Abrolhos*	ND	ND	ND
2008	*Abrolhos*	ND	ND	ND
2009	*Abrolhos*	ND	ND	ND
2010	*Abrolhos*	-0.760	-1.043	-0.703
2011	*Abrolhos*	1.710	1.830	ND
2012	*Abrolhos*	0.769	0.639	ND
2013	*Abrolhos*	0.408	0.357	0.427
2014	*Abrolhos*	-0.104	-0.034	0.395
1995	*Jurien*	ND	ND	ND
1996	*Jurien*	-0.600	-0.652	-0.662
1997	*Jurien*	ND	ND	ND
1998	*Jurien*	ND	ND	ND
1999	*Jurien*	-0.994	-1.038	-1.605
2000	*Jurien*	ND	ND	ND
2001	*Jurien*	-0.966	-1.287	-1.121
2002	*Jurien*	ND	ND	ND
2003	*Jurien*	ND	ND	ND
2004	*Jurien*	ND	ND	ND
2005	*Jurien*	0.283	0.318	0.467
2006	*Jurien*	ND	ND	ND
2007	*Jurien*	ND	ND	ND
2008	*Jurien*	ND	ND	ND
2009	*Jurien*	ND	ND	ND
2010	*Jurien*	-1.074	-0.865	-0.878
2011	*Jurien*	1.731	1.535	1.424

2012	*Jurien*	0.582	0.956	1.120
2013	*Jurien*	0.780	0.895	1.047
2014	*Jurien*	0.258	0.136	0.207
1995	*Rottnest*	-0.452	-0.138	-0.013
1996	*Rottnest*	0.600	0.738	0.949
1997	*Rottnest*	0.289	0.350	0.451
1998	*Rottnest*	0.144	0.262	0.382
1999	*Rottnest*	1.142	1.300	1.444
2000	*Rottnest*	0.166	0.357	0.378
2001	*Rottnest*	-0.390	-0.227	0.093
2002	*Rottnest*	-0.494	-0.278	-0.141
2003	*Rottnest*	-2.586	-2.526	-2.388
2004	*Rottnest*	-0.469	-0.562	-0.471
2005	*Rottnest*	-1.741	-1.827	-1.757
2006	*Rottnest*	0.178	0.173	0.289
2007	*Rottnest*	-0.134	0.049	0.205
2008	*Rottnest*	0.202	-0.267	-0.236
2009	*Rottnest*	-0.049	-0.161	-0.021
2010	*Rottnest*	-0.259	-0.557	-0.527
2011	*Rottnest*	1.962	1.821	1.898
2012	*Rottnest*	1.160	1.043	1.136
2013	*Rottnest*	1.051	0.830	0.783
2014	*Rottnest*	-0.318	-0.382	-0.378
2015	*Rottnest*	ND	ND	-2.076

Supplementary table S6. Number of data points obtained to calculated averages, and anomalies of temperature at each year location and depth.

Depth	*Year*	*Abrolhos*	*Jurien*	*Rottnest*
**15 m**	1995	2	0	10
	1996	7	8	13
	1997	0	0	8
	1998	0	0	14
	1999	1	1	11
	2000	1	1	15
	2001	0	1	12
	2002	0	1	2
	2003	0	0	9
	2004	0	0	9
	2005	1	1	9
	2006	0	0	11
	2007	0	0	5
	2008	0	0	8
	2009	0	0	22
	2010	1	3	16
	2011	1	3	20

	2012	1	4	16
	2013	1	3	15
	2014	4	4	14
	2015	0	0	0
**25 m**	1995	2	0	9
	1996	7	8	13
	1997	0	0	8
	1998	0	0	14
	1999	1	1	11
	2000	1	1	15
	2001	0	1	12
	2002	0	1	2
	2003	0	0	8
	2004	0	0	9
	2005	1	1	9
	2006	0	0	11
	2007	0	0	5
	2008	0	0	9
	2009	0	0	24
	2010	1	4	19
	2011	1	3	27
	2012	1	3	20
	2013	1	2	22
	2014	4	4	16
	2015	0	0	0
**40 m**	1995	2	0	9
	1996	6	8	12
	1997	0	0	8
	1998	0	0	13
	1999	1	1	11
	2000	1	1	15
	2001	0	1	12
	2002	0	1	2
	2003	0	0	7
	2004	0	0	9
	2005	1	1	9
	2006	0	0	11
	2007	0	0	5
	2008	0	0	9
	2009	0	0	24
	2010	1	3	19
	2011	0	2	27
	2012	0	2	20
	2013	1	3	22
	2014	3	4	18

2015	0	0	1

References

Integrated Marine Observing System (2015), MARVL3 - Australian shelf temperature data atlas. f9b50e93-df47-4317-8f1f-f3ed2fed7093. Data accessed at (https://portal.aodn.org.au/)
